# An Unusual Proximal Tibia Physis Injury in an Adolescent

**DOI:** 10.7759/cureus.12052

**Published:** 2020-12-13

**Authors:** Luke S Duggleby, Adam Smith

**Affiliations:** 1 Trauma and Orthopaedics, Royal United Hospital, Bath, GBR

**Keywords:** proximal tibia fracture, child and adolescent

## Abstract

Tibial tuberosity fractures are uncommon but are usually seen in adolescents approaching skeletal maturity. Typically this fracture results from an avulsion of the tibial tuberosity as the powerful quadriceps overcome skeletal strength in passive flexion. We present the case of a 17-year-old female who presented with severe pain in her left knee sustained after stepping off a curb. She had no significant past medical history apart from a raised body mass index (BMI) of 46. Radiographs demonstrated that she had sustained a rare type of physeal injury not previously reported in the literature. This unique fracture developed along the physeal scar but interestingly the anterior cortex remained intact. Closed reduction of this fracture was attempted and the fracture healed uneventfully leaving a slight asymptomatic positive slope on the tibia.

## Introduction

Fractures of the proximal tibia are uncommon injuries but have been reported to occur in predominantly males approaching skeletal maturity with well-developed quadriceps muscles [1-9]. It has been postulated that the tuberosity is an area that is predisposed to avulsion injuries due to the process of ossification that occurs here [9]. Typically these fractures fit a described avulsion type injury with reproducible fracture patterns [1].

The mechanism of injury that causes acute avulsion fracture of the proximal tibial is produced by traction from the patella and a rapid passive flexion of the knee [9]. With increasing energy, the fracture propagates posteriorly along the physis to exit at the posterior aspect of the tibia effectively avulsing the tibial plateau [2]. This case report demonstrates a unique injury that does not fit the previously described mechanism or fracture pattern.

In this report, we present a rare case of a skeletally mature female who was fractured through the weakness in the physeal scar. We discuss the mechanism of injury and the predisposing factors that likely demonstrate that this fracture, although similar to a tibial tuberosity fracture in many ways, is entirely a different entity.

## Case presentation

A 17-year-old female presented with severe pain in her left knee after stepping off of a curb. The patient described the mechanism of injury as hyperextension. The patient was unable to weight bear after the injury and presented to a local minor injuries unit. She had no significant previous medical history or history of a previous traumatic knee injury. Of note, her body mass index (BMI) was 46.1, with a weight of 130 kg.

Examination of the limb revealed a swollen left knee with a moderate joint effusion. On examination, there was no ligamentous laxity of the anterior cruciate or posterior cruciate ligaments. The patient’s medial collateral and lateral collateral ligaments were intact. No neurological or vascular deficit was demonstrated. Ankle-brachial pressure index of the affected limb was 1.0.

**Figure 1:**
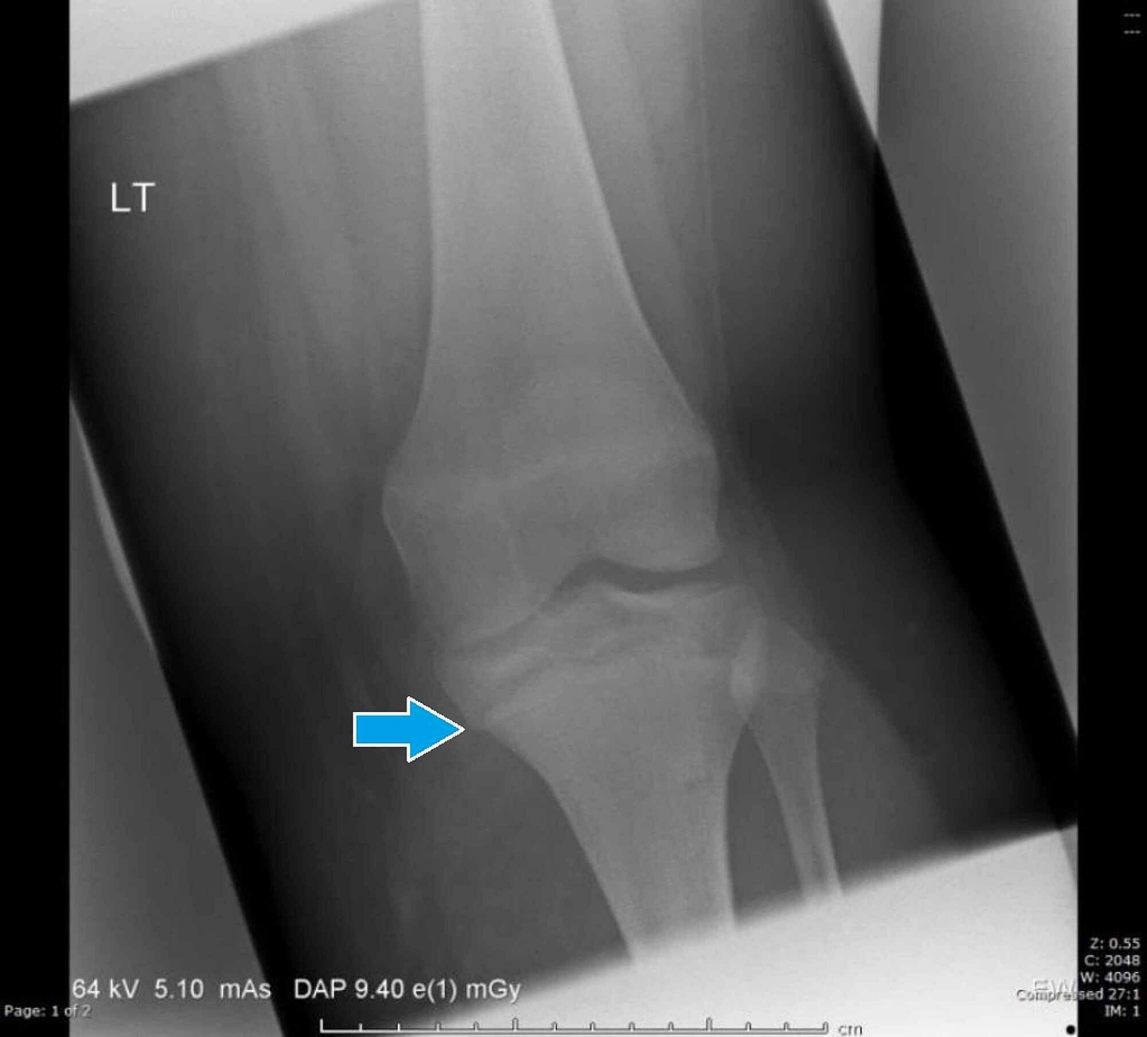
Radiograph of left knee upon initial injury (anterior posterior view)

**Figure 2:**
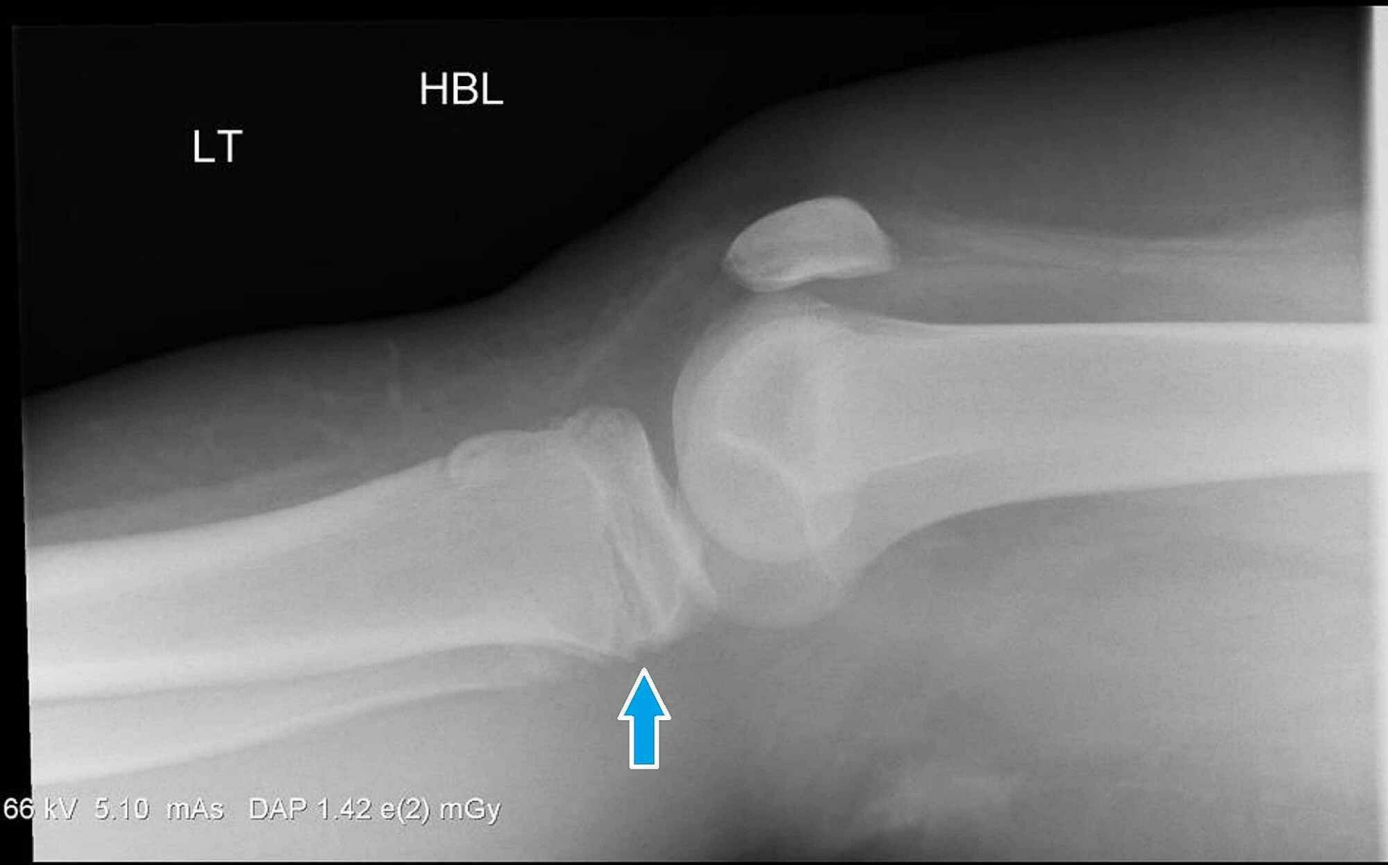
Radiograph of left knee upon initial injury (lateral view)

**Figure 3:**
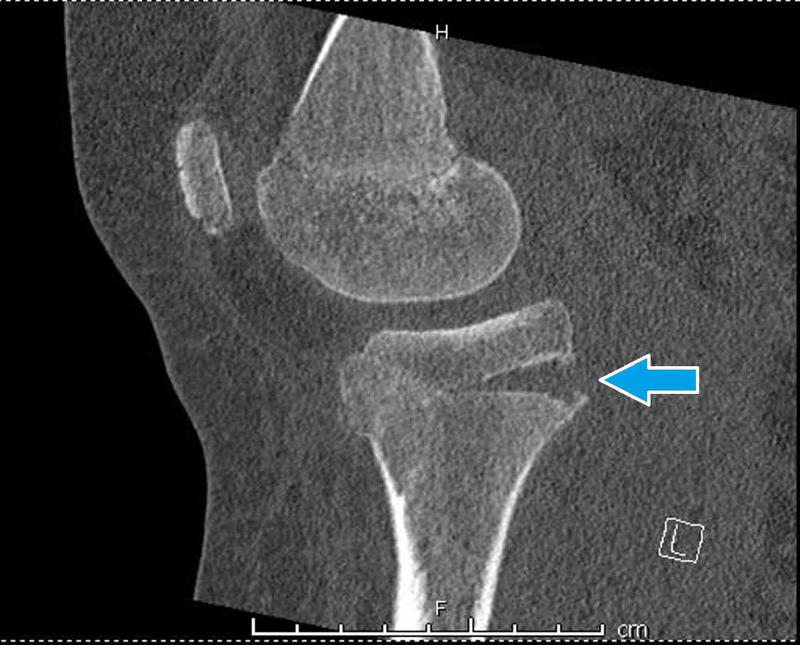
CT of left knee; sagittal view of medial femoral condyle and tibia

Radiographs of the patient’s left knee demonstrated a fracture through the physis of the proximal tibia (Figure 1). The posterior aspect of the physis had opened and was hinging on intact anterior cortex (Figure 2). The lateral femoral condyle additionally demonstrated deformity of the articular surface; this is best demonstrated by the CT in Figure 3. The impaction over the lateral femoral condyle was felt to be caused by abutment of the femur with the tibia in the hyperextended position causing a lateral femoral notch sign [10].

Closed reduction by manipulation of the joint was attempted under general anaesthetic with the knee being flexed. Intra operative imaging showed that unfortunately, this manoeuvre failed to reduce the fracture; the procedure was subsequently abandoned. On balance, further attempts at reduction were felt to carry greater risk due to concerns that this could result in propagation through the intact anterior tibial plateau and tuberosity.

**Figure 4:**
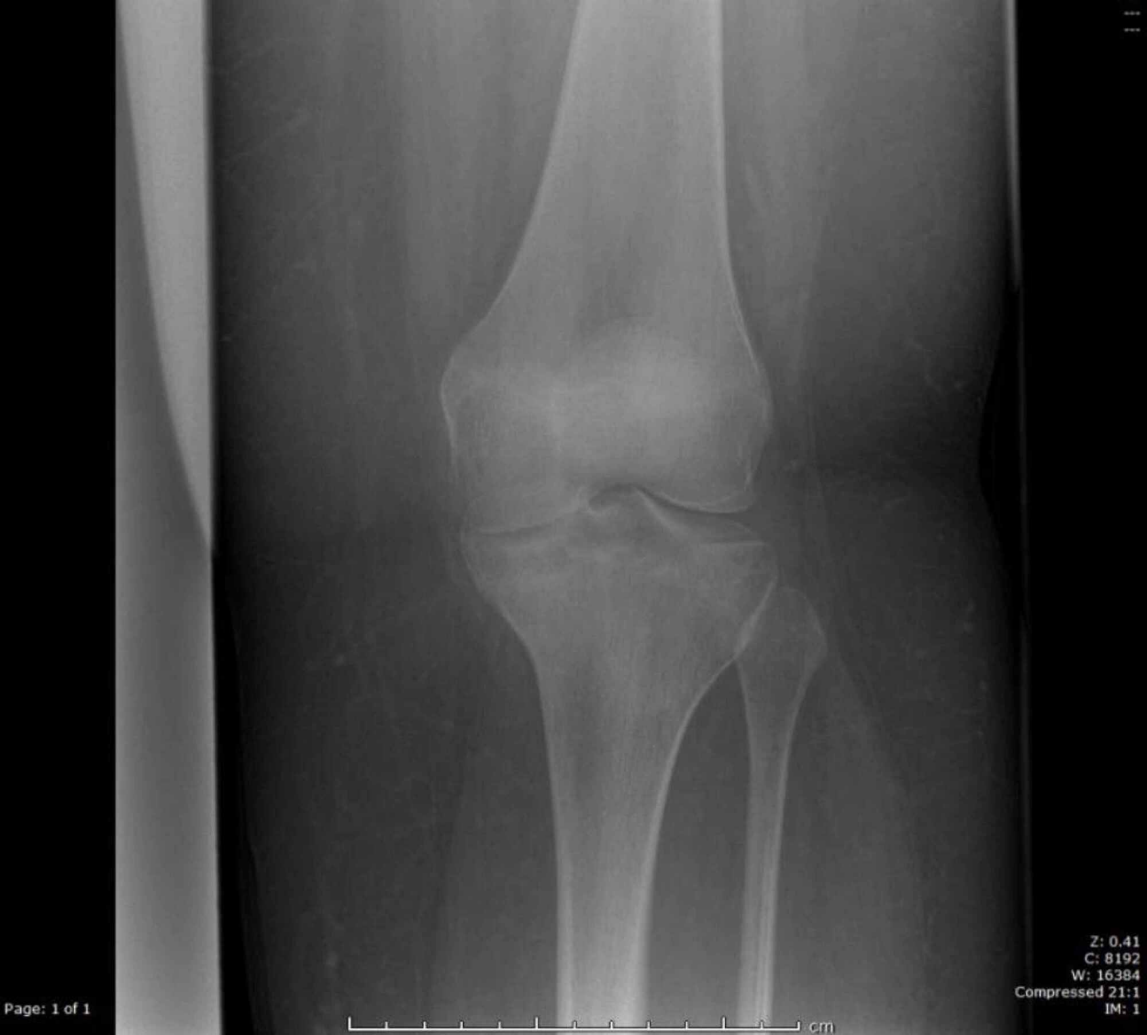
Radiograph of left knee at 12 weeks post injury (anterior posterior view)

**Figure 5:**
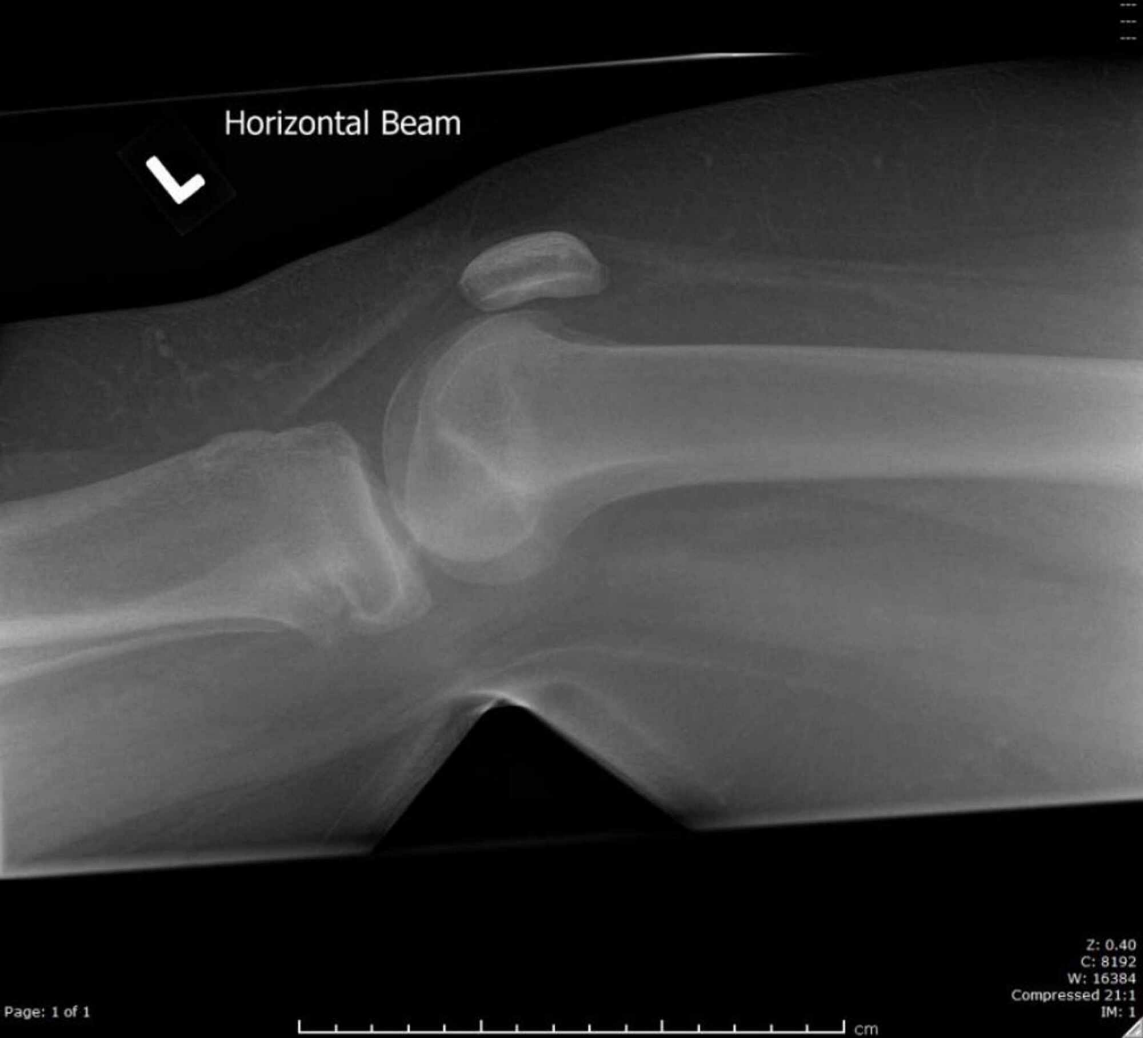
Radiograph of left knee at 12 weeks post injury (lateral view)

This patient was treated in a knee brace with a range of motion from 0 to 90 degrees with partial weight bearing for the first four weeks. After four weeks, full weight bearing was commenced with the brace in full range of motion for a further two weeks. Further follow up at twelve weeks with plain films of the knee revealed fracture union (Figures 4-5).

**Figure 6:**
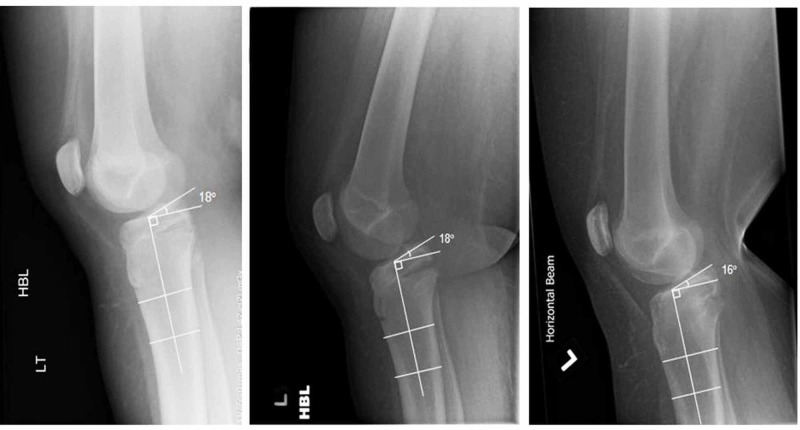
Lateral radiographs at (left to right) initial injury, four weeks post injury and 12 weeks post injury; tibial slope angle measurements displayed

The fracture went on to unite leaving a positive slope of the tibial plateau (Figure 6). The slope of the tibia remained largely unchanged from the initial injury and is illustrated in Figure 6. At first presentation, the tibial slope measured 18 degrees and at final follow up it measured 16 degrees of positive slope. At 12 weeks post injury, the patient had no symptoms of pain, stiffness, or instability of the knee. Examination revealed genu recravatum of 5 degrees and was symmetrical to the contralateral knee. The patient was subsequently discharged from further follow up.

## Discussion

Similar fractures have been described in adolescents though, these typically involve an avulsion of the tibial tuberosity [1-9]. Ogden et al. described fracture of the tibial tubercle which then propagates proximally through the primary ossification center and posteriorly through the tibia [1,9]. During the epiphyseal stage, the secondary ossification center of the proximal tibia and the tubercle join and is present typically complete in girls by age 15 and boys by age 17 [9].

The tibial tuberosity develops from a secondary ossification center and it has been postulated that the combination of osseous and apophyseal changes that occur as fusion across the physis predisposes to injury [9]. Fracture propagation along the physis of the proximal tibia often occurs with significant energy and arises due to the timing and orientation of the physis [2].

Key differences in this case from previously described injuries are the mechanism and the fracture pattern [1-9]. This previously undescribed fracture pattern resulted from a low energy hyperextension injury to the knee. This case is of particular interest since the patient is an adolescent who had almost reached skeletal maturity at the time of injury. As demonstrated in Figure 1, the fracture has occurred through a partially closed physis. The anterior medial portion of the tibial physis remained intact and has acted as an intact hinge with opening of the posterior physis.

In this patient, mechanical factors such as the raised BMI of the patient and slight genu recurvatum have contributed to this fracture pattern. The posterior portion of the tibial plateau has failed under tension.

The fracture went on to unite uneventfully at 12 weeks. A positive slope of the tibial plateau was noted on follow up imaging, as seen in Figures 4-5. Importantly the patient had returned to normal function and if she did become symptomatic in the future, then a corrective osteotomy to reduce the tibial slope would be an option.

## Conclusions

Tibial plateau injuries in adolescents are uncommon injuries and often affect the tibial tuberosity and extensor mechanism of the knee. Due to the orientation of physeal fusion in the proximal tibia, fracture propagation can occur. This case demonstrates a unique type of tibial plateau fracture through the physis of the proximal tibia. The high BMI of the patient was likely a key mechanical factor in causing such a significant injury from a relatively innocuous mechanism. Reduction of this fracture type with closed techniques should be approached with caution to avoid further propagation of the fracture through the tibial tuberosity.
